# Intracranial mesenchymal tumor with (novel) COX14::PTEN rearrangement

**DOI:** 10.1186/s40478-023-01596-9

**Published:** 2023-06-13

**Authors:** Antonio d’Amati, Francesca Gianno, Luciana Scuccimarri, Michele Lastilla, Raffaella Messina, Francesco Signorelli, Domenico Sergio Zimatore, Sabina Barresi, Evelina Miele, Rita Alaggio, Sabrina Rossi, Eugenio Maiorano, Giuseppe Ingravallo, Felice Giangaspero, Manila Antonelli

**Affiliations:** 1 Unit of Anatomical Pathology, Department of Precision and Regenerative Medicine and Ionian Area, University of Bari “Aldo Moro”, Piazza Giulio Cesare 11, Bari, 70124 Italy; 2 Unit of Human Anatomy and Histology, Department of Translational Biomedicine and Neuroscience (DiBraiN), University of Bari “Aldo Moro”, Piazza Giulio Cesare 11, Bari, 70124 Italy; 3 Unit of Anatomical Pathology, Department of Radiology, Oncology and Anatomical Pathology, University La Sapienza, Viale Regina Elena 324, Rome, 00161 Italy; 4 Division of Neurosurgery, Department of Translational Biomedicine and Neuroscience (DiBraiN), University of Bari “Aldo Moro”, Piazza Giulio Cesare 11, Bari, 70124 Italy; 5 Interventional and Diagnostic Neuroradiology Unit, University Hospital Policlinico of Bari, Piazza Giulio Cesare 11, Bari, 70124 Italy; 6 Pathology Unit, Department of Laboratories, Bambino Gesù Children’s Hospital, IRCCS, Piazza Sant’Onofrio 4, Rome, 00165 Italy; 7 Department of Pediatric Onco-Hematology and Cell and Gene Therapy, Bambino Gesù Children’s Hospital, IRCCS, Piazza Sant’Onofrio 4, Rome, 00165 Italy

**Keywords:** CNS tumors, Mesenchymal tumors, COX14, PTEN

## Abstract

Mesenchymal tumors of the central nervous system (CNS) include numerous entities, with different pathological features and biological behavior. Mesenchymal non-meningothelial tumors are rare and comprise neoplasms that are exclusive to the CNS or show peculiar features when occurring in the CNS compared with other sites. Within this group there are three new entities, classified on the basis of specific molecular alterations and included in the 5th edition of the WHO Classification of CNS Tumors: primary intracranial sarcoma; DICER1-mutant; CIC-rearranged sarcoma; intracranial mesenchymal tumor, FET::CREB fusion-positive. These tumors often show variable morphology, making diagnosis very challenging, although the implementation of molecular techniques has led to better characterization and more precise identification of these entities. However, many molecular alterations have yet to be discovered and some recently reported CNS tumors are currently missing an appropriate classification. Herein, we report the case of a 43-year-old man who presented with an intracranial mesenchymal tumor. Histopathological examination showed a wide spectrum of peculiar morphological features and a non-specific immunohistochemical profile. Whole transcriptome sequencing revealed the presence of a novel genetic rearrangement involving *COX14* and *PTEN* genes, which has never been reported before in any other neoplasm. The tumor did not cluster in any defined methylation class of the brain tumor classifier, but resulted in a calibrated score of 0.89 for the methylation class “Sarcoma, MPNST-like”, when analyzed by the sarcoma classifier. Our study is the first to report about this tumor with unique pathological and molecular features, characterized by a novel rearrangement between *COX14* and *PTEN* genes. Other studies are necessary in order to define it as a new entity or as a novel rearrangement involving recently described and incompletely characterized CNS mesenchymal tumors.

## Introduction

Mesenchymal tumors of the central nervous system (CNS) represent a wide group of neoplasms, with different clinic-pathological features and variable biologic behavior. Mesenchymal tumors originate from mesodermal-derived precursor cells, capable of developing into all kind of connective tissues. In the CNS, mesenchymal tumors most commonly arise from the meninges rather than the CNS parenchyma or choroid plexus. Conversely, meningioma represents the most common tumor arising from the meninges [25], and only in rare cases may arise in other sites, such as lung [11] or head and neck [13], but is believed to derive from arachnoid cap cells [27], whose exact origin is still debated whether mesenchymal or not. Differently from meningioma, mesenchymal non-meningothelial tumors are rare. In the 2021 World Health Organization (WHO) Classification of CNS Tumors [36], the mesenchymal non-meningothelial tumors include only those entities occurring exclusively in the CNS, showing peculiar histological or molecular features, or relatively common in the CNS compared with other sites. These neoplasms are subclassified on the basis of their differentiation and comprise a subgroup of tumors with uncertain differentiation [21]. Overall, mesenchymal non-meningothelial tumors of uncertain differentiation often show non-specific histology and immunophenotype, making them difficult to diagnose. The implementation of molecular techniques led to a better understanding of these tumors and to the incorporation of novel entities in the 2021 WHO Classification of CNS tumors, mandatory requiring, in clinical practice, the demonstration of specific molecular features for a more precise diagnosis. However, there is much work to be done, with potential molecular alterations yet to be discovered and recently reported CNS tumors, that are currently missing an appropriate classification. Herein, we report the first case of an intracranial mesenchymal tumor with a genetic rearrangement involving *COX14* and *PTEN* genes, which has never been reported before in any other neoplasm.

## Materials and methods

### Clinical and radiological data

This study was performed with the approval of the Institutional Review Board at the Bari Policlinico Hospital, University of Bari “Aldo Moro”. The patient gave signed informed consent for diagnostic and research analyses. Patient characteristics and clinical data were retrieved from hospital records. The radiological review of magnetic resonance imaging (MRI) was performed by a senior neuroradiologist (DSZ).

### Histology and immunohistochemistry

Hematoxylin-eosin (H&E) stained slides and immunostains were performed according to standard protocols on 4 μm sections of formalin-fixed paraffin-embedded (FFPE) surgically excised specimens, using the following primary antibodies: glial fibrillary acidic protein (GFAP) (1:200, clone 6F2, Dako, Glostrup, Denmark), epithelial membrane antigen (EMA) (1:200, clone GM008; Dako, Glostrup, Denmark), alpha-smooth muscle actin (SMA) (1:300, clone1A4, Dako, Glostrup, Denmark), cytokeratin AE1/AE3 (1:100, clone AE1/AE3, Dako, Glostrup, Denmark), ERG (1:200, clone IR659, Dako, Glostrup, Denmark), STAT6 (1:100, clone YE361, Biocare Medical, Pacheco, CA, USA), S100 (1:2000, polyclonal, Dako, Glostrup, Denmark), synaptophysin (1:150, clone DAK-SYNAP, Dako, Glostrup, Denmark), CD31 (1:50, clone JC70A, Dako, Glostrup, Denmark), CD34 (1:40, clone Qbend10, Dako, Glostrup, Denmark), SOX10 (1:200, clone IHC010, Diagomics, Blagnac, France), H3K27me3 (1:200, polyclonal, Invitrogen, Waltham, MA, USA), desmin (1:200, clone D33, Dako, Glostrup, Denmark), MUC4 (1:200, clone 8G7, Santa Cruz Biotechnology, Santa Cruz, CA, USA), CD99 (1:10, clone 12E7, Dako, Glostrup, Denmark), progesterone receptor (1:100, clone PgR 636, Dako, Glostrup, Denmark), CD68 (1:200, clone PG-M1, Dako, Glostrup, Denmark), melan-A (1:400, clone A103, Dako, Glostrup, Denmark), melanosome (1:400, clone HMB-45, Dako, Glostrup, Denmark), PTEN (1:200, Cell Signaling, clone 138G6) and Ki67 (1:200, clone MIB-1, Dako, Glostrup, Denmark). Automated immunostaining was performed with a Ventana Benchmark XT system (Roche AG, Basel, Switzerland) according to manufacturer instructions. Briefly, after dewaxing and inactivation of endogenous peroxidases (PBS/3% hydrogen peroxide), antibody specific antigen retrieval was performed, sections were blocked and afterwards incubated with the primary antibody. Diaminobenzidine (DAB) was used as the chromogen. Counterstaining for nuclei was performed using Mayer’s hematoxylin. External positive and negative controls were used for all antibodies. Photographs were taken from slides scanned with a Hamamatsu NanoZoomer S60 automatic digital slide scanner (Hamamatsu Photonics, Hamamatsu, Japan), obtaining images of whole stained sections at a resolution of at least 1 pixel per µm. The central pathology review was performed conjointly by two expert neuropathologists (FeG and MA) and a pathologist expert in soft tissue tumors (RA).

### Fluorescence in situ hybridization (FISH)

FISH was performed on 4 μm-thick FFPE tissue sections. *EWSR1* (22q12.2) rearrangement was evaluated using the Empire Genomics *EWSR1* break-apart FISH probe kit (Empire Genomics, New York, NY, USA). The slides were deparaffinized, pretreated and hybridized with denaturated probes. After overnight incubation, the slides were washed, stained with DAPI, mounted with an antifade solution, and then examined on Nikon fluorescence microscope (Nikon, Tokyo, Japan).

### Whole transcriptome sequencing

RNA was extracted from formalin-fixed paraffin-embedded tumour tissue following ReliaPrep™ FFPE Total RNA kit (Promega) and quality and quantity of RNA samples was ascertained with the use of Agilent 2200 Tapestation system (Agilent Technologies). The SureSelect XT HS2 RNA kit (Agilent Technologies) was used to prepare RNA sequencing libraries from 300 ng of total RNA according to the manufacturer’s protocol. Libraries were pooled and sequencing run was performed in paired-end mode using the NextSeQ 550 system (Illumina, San Diego, California) generating at least 30 million reads per sample. Raw reads were aligned to the reference human genome (UCSC-Build38) using STAR (2.5.3a) algorithm. Arriba Fusion, STAR Fusion and DRAGEN RNA (v3.7.5) pipeline were used for fusion detection. The candidate list of potential fusion transcripts was filtered by removing any known false positives or transcripts which were out of frame.

### DNA methylation profiling and copy number analysis

Tumor samples from FFPE tissue specimens including ten scrolls with 10 μm in thickness were utilized, selecting areas with the available tumor cellularity over 70%. Genomic DNA was extracted according to MagPurix FFPE DNA Extraction Kit (Zinexts, Life Science Corporation, New Taipei City, Taiwan) for automatic extraction of genomic DNA. DNA methylation profiling was performed, as previously reported [23, 29], using the Infinium Methylation EPIC (850k) BeadChip (Illumina, San Diego, CA, USA), according to the manufacturer’s instructions. Standard quality controls confirmed adequate tumor purity/quality, bisulfite conversion, and DNA quality. Raw methylation intensity data files (IDATs) were uploaded to either version 11b4 or 12.5 of the DKFZ/Heidelberg CNS tumor methylation Classifier or version 12.2 of the Sarcoma Classifier (https://www.molecularneuropathology.org) and reports were produced as shown by Capper et al. [5] and Koelsche et al. [15], respectively. Raw methylation data were instrumental to determine copy number variation plots (CNV) that were generated for the reported case, as described [5]. Graphical visualization of structural rearrangements was performed by Integrative Genomic Viewer (IGV) [29].

## Case presentation

### Clinical history and radiological characteristics

**Fig. 1:**
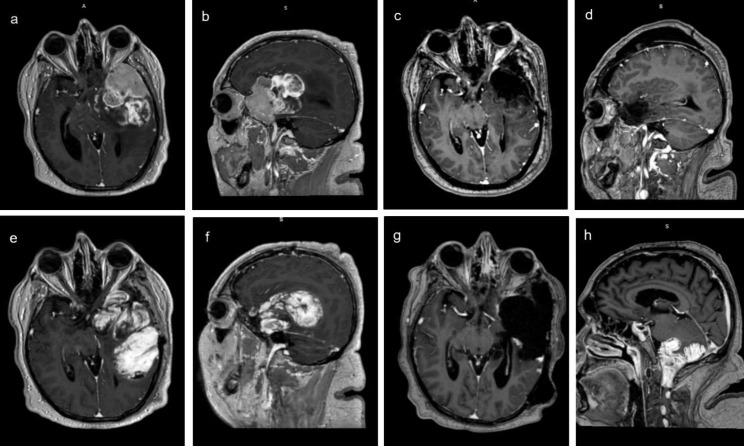
Radiological features. A voluminous tumour in the left temporal pole, extending superiorly to fronto-basal and fronto-lateral regions, deeply to the insula and posteriorly till the supramarginal gyrus was discovered (**a, b**). The tumour was initially interpreted as atypical meningioma with peripheral intratumoral degenerative cysts. The tumour was completely resected with no signs of residue; brain tissue’s compression resolved (**c, d**). A two months post-surgery MRI control demonstrated a huge left fronto-temporal local tumoral recurrence (**e, f**). Three months MRI control after a second intervention showed only a small local recurrence on the posterior margin of the surgical cavity but a diffuse leptomeningeal tumoral spread on the inferior surface of cerebellum, around the brainstem and around the spinal cord (**g, h**)

A 43-year-old male was admitted to the Division of Neurosurgery, Department of Translational Biomedicine and Neurosciences (DiBraiN), at the University “Aldo Moro” of Bari, for speech disorder, mild right hemiparesis and visual impairment. Neurological examination also revealed drowsiness, confusion and non-fluent semantic aphasia. In the previous two years, the patient showed behavioral and mnesic disorders and, after receiving a clinical diagnosis of reactive depression, was treated with psychotherapy. Brain MRI showed a voluminous tumor in the left temporal pole, superiorly extending to fronto-basal and fronto-lateral regions, deeply to the insula and posteriorly to the supramarginal gyrus. The lesion presented strong and inhomogeneous enhancement after gadolinium administration, with an anterior solid portion and areas of cystic degeneration in the posterior peripheral side. Relevant mass effect with midline shift and perilesional oedema was observed. The tumour was initially interpreted as atypical meningioma with intratumoral degenerative and necrotic cysts (Fig. 1a, b). Hence, the patient underwent a surgical procedure with a fronto-orbito-zygomatic (FOZ) approach, conducted with the aid of neuronavigation system and neurophysiological monitoring, to reach a total resection and a supramarginal resection of the mass as far as possible, due to the extension of the lesion in highly functional areas. Post-operative MRI showed a total resection of the lesion (Fig. 1c, d). Afterward, the patient started neuromotor rehabilitation and a combined chemotherapy and radiotherapy regimen. However, the tumor relapsed only two months after surgery. The patient’s consciousness and the behavioral and mnesic disturbances worsened again and brain MRI showed a huge recurrence of the lesion, which completely filled the previous surgical cavity (Fig. 1e, f). A surgical second look was performed. The respiratory condition worsened after surgery and the patient turned out COVID-19 positive. Brain MRI performed twenty days after surgery and after recovery from COVID, showed a nodular residue/recurrence on the posterior margins of the surgical cavity. A huge CSF collection, pseudomeningocele-type, was treated with a subgaleal-peritoneal shunt. After a good neurological recovery and in the shortest time possible, the patient began a non-conventional approach of radiotherapy with Volumetric Modulated Arc Radiation Therapy (VMAT) of Arc 1–6 X VMAT and Arco 2–10 X VMAT on the surgical cavity for a total dose of 60 Gy in 30 fractions (DTF: 2 Gy/die) carried out in 38 days. Immediately after the stop of radiation therapy, the patient displayed a worsening of neurological symptoms and general condition. Brain and spine MRI showed a dramatic leptomeningeal spread of the tumour, which involved the inferior surface of cerebellum, wrapped the brainstem and disseminated along the spinal cord. The recurrence on the margin of the irradiated cavity, on the other hand, was not significantly increased (Fig. 1g, h). Shortly after, the patient deceased. Hence, considering histological peculiar features, aggressive course and rapid progression of the tumor, all samples were sent for central pathological review to the Division of Anatomical Pathology, Department of Radiology, Oncology and Anatomic Pathology, at the University “La Sapienza” of Rome.

### Histological features

**Fig. 2:**
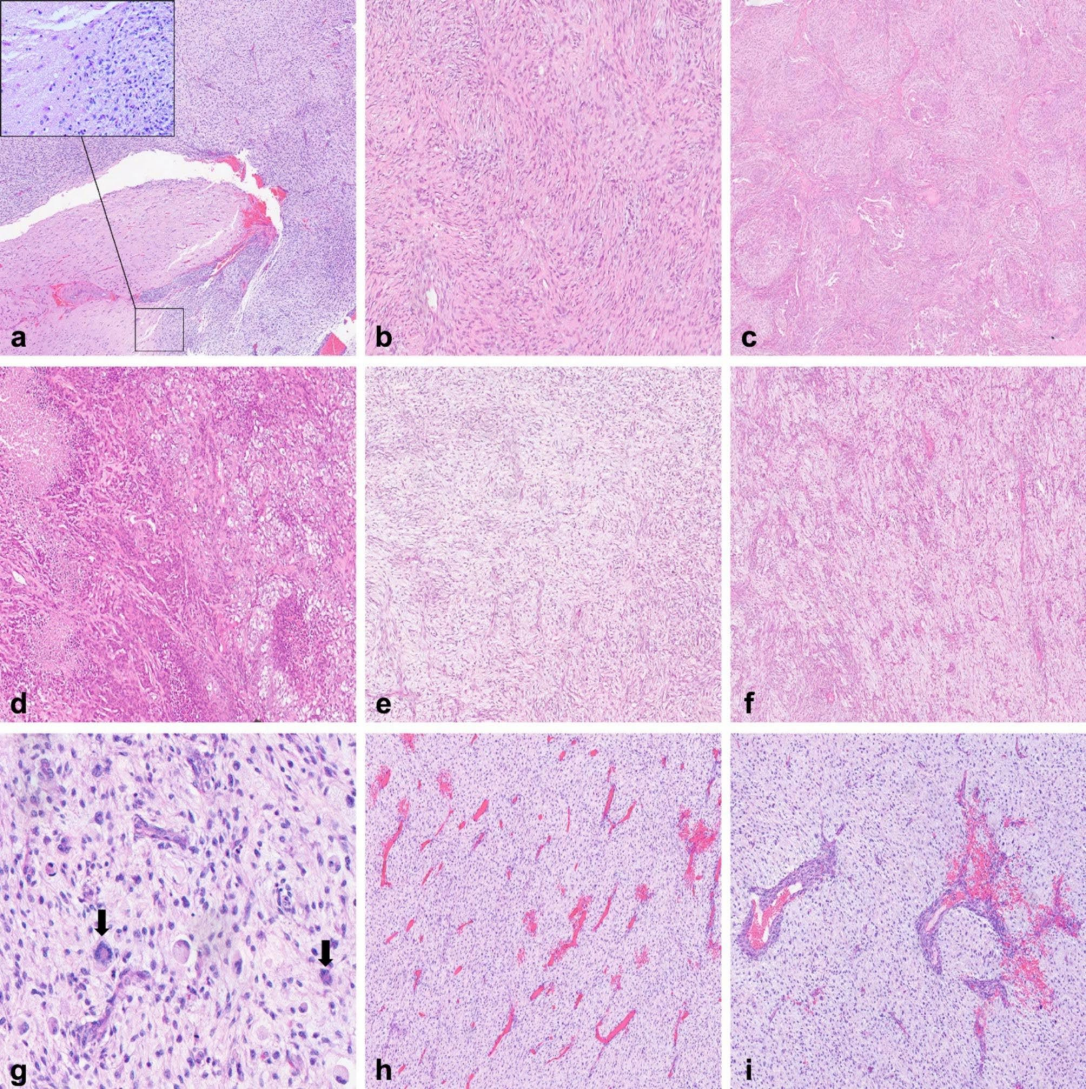
Histological findings. The tumor showed circumscribed borders with focal areas of irregular tumor-brain interface, suggestive of brain invasion (inset, higher magnification), and tumor nests extending along the Virchow-Robin spaces (**a**). The hypercellular spindle areas appeared as short irregular fascicles of elongated cells, immersed in a dense collagenous stroma (**b**). The hypercellular epithelioid areas showed a predominant lobular architecture (**c**). In other adjacent areas, epithelioid cells were organized in sheet-like and cord-like structures, with clearer cytoplasm and multiple necrotic foci (**d**). The hypocellular areas were composed of a myxoid stroma with immersed spindle or stellate cells (**e**), often showing an angiocentric arrangement (**f**). In some myxoid areas there were more evident cytologic atypia and numerous *daisy-like* multinucleated elements (indicated by arrows), characterized by intracytoplasmic eosinophilic globules encircled by multiple nuclei (**g**). A rich vascular component was diffusely present, prevalently composed of small thin-walled vessels (**h**), and focally of larger vessels with thickened walls (**i**)

Microscopically, the tumor was almost circumscribed and demonstrated a vague nodular architecture, with focal areas showing an irregular tumor-brain interface, suggestive of brain invasion, together with tumor nests extending along the Virchow-Robin spaces (Fig. 2a). It showed a wide spectrum of morphological features, being composed of hypercellular and hypocellular areas. In the hypercellular areas, tumor cell morphology included spindle cells and epithelioid cells. Spindle cells were monotonous and mildly atypical, with oval-to-spindled nuclei, speckled chromatin and eosinophilic cytoplasm. They were arranged in short irregular fascicles, immersed in a dense collagenous stroma (Fig. 2b). Epithelioid cells were more pleomorphic and atypical, with round-to-oval nuclei, and larger amount of clear or eosinophilic cytoplasm. They showed a predominant lobular architecture, vaguely recalling pseudo-meningothelial aspects (Fig. 2c). Adjacent to lobular areas, epithelioid cells also showed sheet-like and reticular cord-like structures (Fig. 2d). The hypocellular areas were composed of mildly atypical spindle cells, surrounded by an abundant collagenous stroma, or stellate and round-to-oval cells, immersed in a largely myxoid stroma (Fig. 2e). These cells were arranged in short cords or as single cells and showed a discrete angiocentric pattern (Fig. 2f). In some myxoid areas, tumor cells showed abrupt transition to hypercellular foci with severe cytologic atypia, characterized by the presence of multinucleated elements, with intracytoplasmic eosinophilic globules (Fig. 2g). Throughout the tumor there was a rich vascular network, prevalently composed of small thin-walled vessels (Fig. 2h), and sometimes showing larger vessels with thickened walls (Fig. 2i). Scarce perivascular lymphoplasmacytic infiltrates and hemorrhagic spots were present in some central areas, but not at the periphery of the tumor. Necrosis was easily observable throughout the tumor, particularly in epithelioid hypercellular areas. Mitotic index was quite variable, being lower in areas composed of monotonous spindle cells fascicles (up to 7/10 high power fields, i.e. HPF), higher in epithelioid areas (up to 12/10 HPF) and reaching a peak in myxoid areas with atypical cells (up to 23/10 HPF).

### Immunohistochemical features

**Fig. 3:**
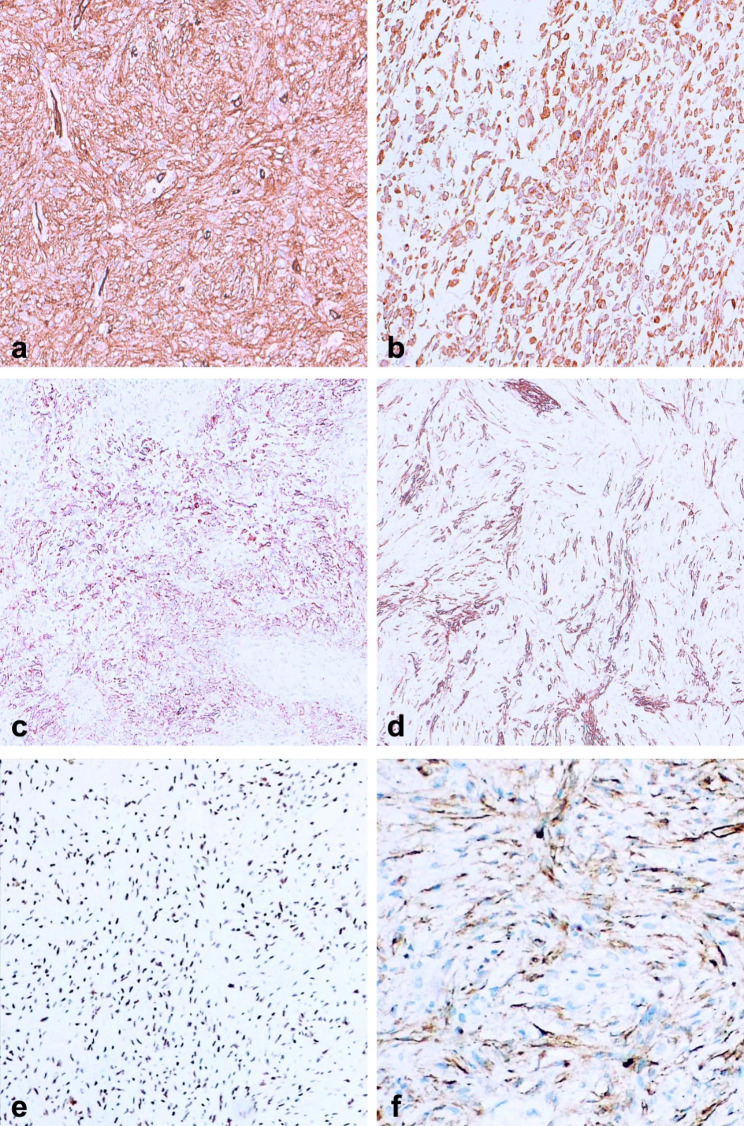
Immunohistochemical results. The tumor resulted diffusely and strongly positive for CD34 (**a**) and MUC4 (**b**). The tumor cells were also positive for EMA and SMA, but in a patchier manner (**c, d**). The H3K27me3 expression was retained in the nuclei of tumor cells (**e**). Conversely, tumor cells showed PTEN loss of expression, particularly in the nuclei, while appeared retained in endothelial cells (**f**)

Tumor cells showed strong and diffuse positivity for CD34 (Fig. 3a) and MUC4 (Fig. 3b). The EMA and SMA immunostains showed patchy positivity, with the latter being particularly expressed in perivascular tumor cells of angiocentric pattern foci (Fig. 3c, d). Very weak and focal immunostaining was observed for CD99 and desmin. The GFAP, cytokeratin AE1/AE3, CD31, ERG, STAT6, S100, SOX10, melan-A, melanosome/HMB-45, synaptophysin, progesterone receptor, CD68 immunostains were negative. The H3K27me3 immunostain showed retained nuclear expression (e). PTEN immunostain showed loss of expression, particularly in the nuclei of neoplastic cells, with retained expression in internal positive controls (i.e. endothelial cells) (f). The proliferation labeling index, evaluated with the Ki67 immunostain, was high, showing a mean value of 25%. However, in some myxoid foci, the rate was up to 40%.

### Fluorescence ***in situ*** hybridization

After central pathology review, because of peculiar histological and immunohistochemical features, a diagnosis of intracranial mesenchymal tumor, FET::CREB fusion-positive was suspected. However, FISH assay showed no rearrangements for *EWSR1* (22q12.2).

### RNAseq

**Fig. 4:**
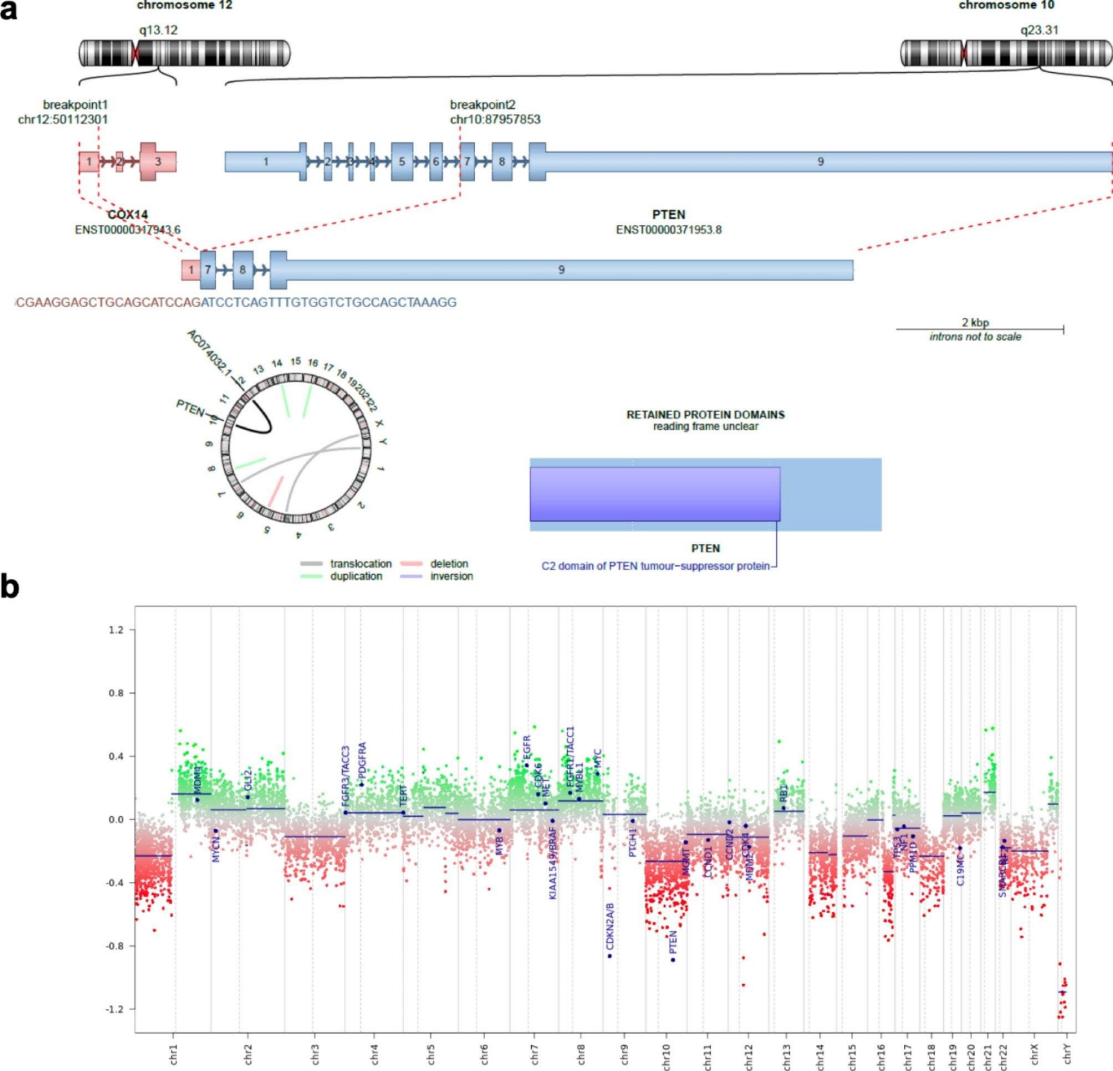
Molecular findings. RNAseq revealed a reading frame unclear transcript between *COX14*-*PTEN*, with disruption of the C2 domain of PTEN protein (**a**). The copy number variation profile obtained from DNA methylation analysis (**b**)

A total of 31 million 151 bp paired-end read pairs were generated. The sequencing data were mapped to the human reference gene set RefSeq (hg38) using STAR (2.5.3a) algorithm. RNASEQ analysis reveals a novel chromosome rearrangement result from breakage involving chr12 (q13.12) and chr10 (q23.31) with subsequent re-union between *COX14* exon1 and *PTEN* exon 7 leading a fusion transcript with an unclear reading frame. With this translocation, PTEN loses the 5’ PDB and phosphatase domains and the 3’ C2 domain is broken (Fig. 4a).

### CNV

Copy number variation analysis showed several structural alterations including chromosome 1p loss and 1q gain, chromosome 10, 14, 16q loss, gain of chromosome 7 (*EGFR*), 8 (*MYC*), and 21. In particular deletion in homozygosis of *CDKN2A/B* and *PTEN* genes were present (Fig. 4b).

### DNA methylation profile

According to the brain tumor classifiers the tumor did not cluster in any defined methylation class of the v11b4 (calibrated scores > 0.3), while reached the calibrated score of 0.69 in the in the methylation class “Malignant peripheral nerve sheath tumour” - MPNST_TYP, of the v12.5. When analyzed by the sarcoma classifier, the sample clustered within the methylation class “Sarcoma, MPNST-like” – SARC_MPNST_LIKE, with a calibrated score of 0.89.

## Discussion and conclusions

Herein, we describe an unusual mesenchymal tumor occurring intracranially in a 43-year-old male, characterized by aggressive course, peculiar morphological features and showing a genetic rearrangement, involving chromosome chr12 (q13.12) and chr10 (q23.31) with loss of PTEN expression. Because of the radiological and histological features, the initial interpretation was of anaplastic meningioma (Grade 3, according to 2021 WHO Classification of CNS Tumors). In our case, tumor cells showed patchy positivity for EMA, similarly to meningiomas, and negativity for progesterone receptor. However, tumor cells also showed diffuse positivity for CD34 and MUC4, patchy positivity for SMA, and focal positivity for CD99 and desmin, suggesting a wide spectrum of differential diagnoses. Mesenchymal non-meningothelial tumors are rare and include many entities, classified on the basis of their differentiation. Solitary fibrous tumors (SFTs) are usually dural based neoplasms, characterized by *NAB2* and *STAT6* fusion [6, 20]. SFT has a wide histological spectrum. Immunohistochemically, CD34 is typically positive, although its expression is reduced in higher grades [26]. Other markers, such as EMA, progesterone receptor, desmin and SMA may be focally expressed. As a consequence of NAB2::STAT6 fusion, SFT usually shows diffuse and intense STAT6 nuclear expression [14, 30]. The case described in our study is a dural neoplasm, showing a wide morphological spectrum and CD34 immunohistochemical positivity. Conversely, STAT6 negativity and the absence of NAB2::STAT6 fusion, as confirmed by RNA sequencing, excluded the diagnosis of SFT. On the basis of the clinico-pathological features, we also excluded other mesenchymal non-meningothelial tumors and focused our attention on the subgroup of tumors with uncertain differentiation. Ewing sarcoma was also excluded, considering the different clinical, histological and molecular aspects. In the 5th edition of the WHO Classification of CNS Tumors, among the subgroup of tumors of uncertain differentiation, there are other three newly recognized entities: primary intracranial sarcoma, DICER1-mutant; CIC-rearranged sarcoma; intracranial mesenchymal tumor, FET::CREB fusion-positive. Primary intracranial sarcoma, DICER1-mutant, has been firstly described by Koelsche et al. [15]. All these tumors were characterized by rhabdomyoblastic differentiation and *DICER1* inactivating mutations. Afterward, other studies better characterized this entity, describing various histological and immunohistochemical aspects [1, 19]. CIC-rearranged sarcoma more commonly occurs outside the CNS, but some cases have been observed also in the brain [2]. Some of these tumors were previously included in the wide category of CNS-PNETs, which has been now eliminated [33]. Similarly to Ewing sarcoma, it is composed of CD99-positive undifferentiated cells, but with a more patchy pattern of expression. Molecularly, they are characterized by the presence of a fusion of *CIC* with various partners [17]. Intracranial mesenchymal tumor, FET::CREB fusion-positive has been introduced in the 2021 WHO Classification of CNS tumors as a provisional entity [36]. This tumor has been firstly described by Kao et al. [10], who identified a group of intracranial myxoid mesenchymal tumors, histologically resembling myxoid variant of angiomatoid fibrous histiocytoma (AFH) and characterized by fusions of *EWSR1* with *CREB* family transcription factors (*CREB1, CREM* or *ATF1*). Soon after, other studies [3, 31, 34] identified similar cases, reporting variable morphological features and novel fusions involving *FUS*, another *FET* family gene, instead of *EWSR1*. Histologically, they demonstrate a wide morphological spectrum. The immunohistochemical profile is also variable, but they most commonly show positivity for EMA, CD99 and desmin. Because of the broad histological and immunohistochemical spectrum, the demonstration of FET::CREB family fusions is an essential criterion for the diagnosis. After central pathology review, a possible diagnosis of FET::CREB fusion-positive intracranial mesenchymal tumor was considered. FISH assay revealed no *EWSR1* rearrangements and RNA sequencing excluded other FET::CREB family fusions. Furthermore, DNA methylation profile found no matches with other known entities. The closer match in CNS tumors classifier v12.5, with 0.69 calibrated score, was malignant peripheral nerve sheath tumor (MPNST), but negativity for S100 and SOX10, along with preserved H3K27me3 nuclear expression, suggested this diagnosis as improbable [9]. However, when analyzed by the sarcoma classifier, the tumor clustered within the methylation class “Sarcoma, MPNST-like”, with a calibrated score of 0.89. Such class has a provisional name and is based on tumors with morphological features of malignant peripheral nerve sheath tumor, but with retained expression of H3K27me3 [28]. This subgroup has been described by Röhrich et al. In their study, they identified 6 cases (5 sporadic, 1 NF1-associated), all with spinal or paravertebral localization and with a median age of 50 years (age range 10–77). All of them were characterized by retained H3K27 trimethylation marker, distinguishing them from MPNSTs. Copy number alterations were numerous with frequent losses on chromosome arms 3q (> 80%), 5q (> 80%), 9p (100%) and 17q (> 70%). Copy number analysis showed a high frequency of CDKN2A/B homozygous deletion on chromosome arm 9p (> 40%). Further molecular features of this class included mutations in NF1. Some of copy number alterations seemed to be shared with MPNSTs, but others were more frequently encountered in this class, such as 3q loss, 5p gain and *CDKN2A* homozygous deletion. In our case, CNV analysis showed several structural alterations. In particular, *CDKN2A* deletion, as observed in MPNST-like tumors, and loss of *PTEN* as a possible consequence of the genetic imbalance due to the above-mentioned rearrangement. Searching in literature and in the ChimerDB 4.0, a comprehensive and updated database of fusion genes (available online at http://www.kobic.re.kr/chimerdb/) [8], we found no data regarding this specific rearrangement. Instead, the two genes, *COX14* and *PTEN*, are independently involved, with other genes as fusion partners, in rare cases of gliomas and mesenchymal tumors. The *COX14* gene is located on the long arm of chromosome 12 and encodes for a core protein component of the enzyme MITRAC (mitochondrial translation regulation assembly intermediate of cytochrome c oxidase complex), which is required for the proper assembly of complex IV of the mitochondrial respiratory chain [22]. *COX14* variants have been associated with complex IV deficiency, characterized by different phenotypes, such as myopathy, cardiomyopathy, liver dysfunction and mental retardation [35]. Even though high levels of mRNA have been reported in different types of malignant neoplasms, particularly in prostatic adenocarcinoma [16], the role of *COX14* in cancer pathogenesis is still unclear. The *PTEN* gene is located on the long arm of chromosome 10 and encodes for PTEN protein (phosphatase and tensin homolog), an enzyme with phosphatase activity and crucial role as tumor suppressor. In fact, its loss of function is frequently observed in both heritable and sporadic neoplasms [18]. In our case, *PTEN* rearrangement showed disruption of C2 domain, suggesting PTEN loss of function, as demonstrated by its loss of expression in immunohistochemistry, and a pathogenic role in the tumor development. In a study by Williams et al. [37], *PTEN* gene has been found altered in some cases of high-grade meningiomas. In this study, *PTEN* alterations were mainly represented by mutation or focal gene deletions, with only one case showing a *PTEN-TMEM38A* rearrangement. Additionally, *PTEN* mutations were found to be more frequently associated with a WHO grade 3 than grade 2 or 1. *PTEN* is rarely mutated in MPNST [24], but in two studies MPNST showed reduced *PTEN* expression compared to neurofibroma [4, 7]. Interestingly, the lack of both *NF1* and *PTEN* led to accelerated neurofibroma development and favored progression from low-grade to high-grade PNSTs in a transgenic murine model [12]. However, *PTEN* has been found downregulated, with variable frequency, also in other sarcoma subtypes. These findings highlight its important role as tumor suppressor, but its prognostic and predictive role are still controversial. Only a small percentage of PTEN-deficient tumors have inactivating mutations, while PTEN function can also be lost through non-genomic mechanisms, including protein-protein interactions, epigenetic and transcriptional silencing and post-translational modifications [32].

The case reported in our study showed non-specific clinical, morphological and immunohistochemical features. On the basis of the DNA methylation profile only, it is difficult to include our case in MPNST-like group with certainty. Nonetheless, in this instance, our case would be the first intracranial MPNST-like sarcoma reported in literature. Additionally, whole transcriptome sequencing revealed a novel chromosome rearrangement, involving *COX14* and *PTEN* genes. This rearrangement has never been reported before in any other neoplasm and, as suggested by the loss of PTEN expression in immunohistochemistry, may play a fundamental pathogenic role in the development of this tumor, because of PTEN loss of function. However, the functional consequence of this novel rearrangement needs to be furtherly studied, because we only found a fusion transcript with an unclear reading frame. Hence, it may not represent a fusion protein, but more probably a rearrangement truncating *PTEN* with loss of the remaining allele. Moreover, as often seen in fusion-driven sarcomas, this rearrangement is accompanied by other alterations, such as *CDKN2A* homozygous deletion and other copy number modifications. Considering the unique clinico-pathological and molecular features, it is not to be excluded that this tumor might also represent a novel entity characterized by COX14::PTEN rearrangement as molecular hallmark, but in absence of other data this hypothesis is premature. On the other hand, this rearrangement may also be a characteristic molecular feature of MPNST-like sarcomas, or, alternatively, it might be present only in intracranial localization of this still uncertain entity. Nevertheless, our study currently represents the first and only case showing this rearrangement, whose pathogenic and diagnostic roles have yet to be fully understood and characterized. Other studies reporting cases with similar molecular and clinico-pathological features are indubitably needed whether to possibly consider it as a new entity or as a novel rearrangement involving CNS mesenchymal tumors, that have already been described and classified.

## Data Availability

The data analysed during the current study are available from the corresponding author on reasonable request.
